# The Role of *Helicobacter pylori* Outer Membrane Proteins in Adherence and Pathogenesis

**DOI:** 10.3390/biology2031110

**Published:** 2013-08-27

**Authors:** Mónica Oleastro, Armelle Ménard

**Affiliations:** 1 Department of Infectious Diseases, National Institute of Health Dr Ricardo Jorge, 1649-016 Lisbon, Portugal; E-Mail: monica.oleastro@insa.min-saude.pt; 2 INSERM U853, Infection à Helicobacter, inflammation et cancer, Bordeaux 33076, France; 3 Laboratoire de Bactériologie, Université de Bordeaux, Bordeaux 33076, France

**Keywords:** bacterial adherence, adhesin, receptor, virulence, gastric disease, outer membrane protein, Lewis antigen

## Abstract

*Helicobacter pylori* is one of the most successful human pathogens, which colonizes the mucus layer of the gastric epithelium of more than 50% of the world’s population. This curved, microaerophilic, Gram-negative bacterium induces a chronic active gastritis, often asymptomatic, in all infected individuals. In some cases, this gastritis evolves to more severe diseases such as peptic ulcer disease, gastric adenocarcinoma, and gastric mucosa-associated lymphoid tissue lymphoma. *H. pylori* has developed a unique set of factors, actively supporting its successful survival and persistence in its natural hostile ecological niche, the human stomach, throughout the individual’s life, unless treated. In the human stomach, the vast majority of *H. pylori* cells are motile in the mucus layer lining, but a small percentage adheres to the epithelial cell surfaces. Adherence to the gastric epithelium is important for the ability of *H. pylori* to cause disease because this intimate attachment facilitates: (1) colonization and persistence, by preventing the bacteria from being eliminated from the stomach, by mucus turnover and gastric peristalsis; (2) evasion from the human immune system and (3) efficient delivery of proteins into the gastric cell, such as the CagA oncoprotein. Therefore, bacteria with better adherence properties colonize the host at higher densities. *H. pylori* is one of the most genetically diverse bacterial species known and is equipped with an extraordinarily large set of outer membrane proteins, whose role in the infection and persistence process will be discussed in this review, as well as the different receptor structures that have been so far described for mucosal adherence.

## 1. Introduction: *Helicobacter pylori*—A Successful Pathogen

About 30 years ago, Barry Marshall and Robin Warren reported the isolation and culture of a spiral bacterial species from the human stomach [1]. *Helicobacter pylori* previously named *Campylobacter pyloridis* is a curved microaerophilic Gram-negative bacterium, 2 to 4 μm long with a diameter of 0.5 to 1 μm. This spiral-shaped bacillus is highly motile due to its unipolar bundle of two to six sheathed flagella and it colonizes the mucus layer of the gastric epithelium of humans. The bacterium migrated out of Africa along with our ancestors and has been intertwined with our species for at least two hundred thousand years [2]. While not as deadly as some bacteria, *H. pylori* is the most successful pathogen in human history since it infects up to half of the world’s population. The prevalence of *H. pylori* infection is the highest in developing countries (up to 100%) where risk factors are mainly low socioeconomic conditions including large family size, while decreasing in Western countries (10%–60%). The most common routes of *H. pylori* transmission are oral-to-oral and fecal-to-oral contact. Parents and siblings seem to play a major role in transmission. 

Acquisition of *H. pylori* infection occurs predominantly in childhood, with more severe gastroduodenal diseases appearing mostly during adulthood. Although *H. pylori* infection always causes a chronic active gastritis, the majority of infected individuals (~85%) remain asymptomatic throughout life. In some cases, this gastric inflammation may evolve toward more severe diseases such as duodenal or gastric ulcers, gastric mucosa-associated lymphoid tissue (MALT) lymphoma or non-cardia gastric adenocarcinoma. *H. pylori* infection was classified as a type I carcinogen in 1994 by the International Agency for Research on Cancer (World Health Organization) [3]. Indeed, *H. pylori* infection is the main risk factor in up to 92% of gastric cancers [4] and this cancer is the fourth most common and second most deadly cancer worldwide, with approximately 740,000 deaths per year. Studies have also associated *H. pylori* infection with diverse extragastric nonmalignant diseases [5]. 

In the absence of an effective vaccine, antibiotic treatment cures peptic ulcer disease and gastric MALT lymphoma permanently (when the treatment is given before the MALT lymphoma becomes autonomous) while gastric adenocarcinoma is difficult to prevent unless it is found at an early stage [6]. *H. pylori* infection can be difficult to treat and requires the combined intake of a proton pump inhibitor with amoxicillin and one of two antibiotics, clarithromycin or metronidazole [7]. More virulent *H. pylori* isolates harbour numerous well-known adhesins (BabA/B, SabA, AlpA/B, OipA and HopZ), the vacuolating cytotoxin VacA and the oncoprotein CagA. The *cagA* gene belongs to the *cag* pathogenicity island (PAI), which encodes a type IV secretion system (T4SS), a needle-like pilus device that delivers effector proteins such as the CagA oncoprotein into the cytoplasm of gastric epithelial cells (for review see [8] and references cited therein).

The adherence of *H. pylori* to the mucus layer of the gastric epithelium plays an important role in the initial colonization and persistence of the bacteria in the human stomach during decades or for an entire lifetime. *H. pylori* colonization of the stomach elicits humoral and cellular immune responses which in most cases do not result in bacterial clearance. The bacterium has developed a unique set of factors, actively supporting its successful survival and persistence in its natural hostile ecological niche, the human stomach, throughout the individual’s life, unless treated. The bacterium is one of the most diverse and variable bacterial species known, constituting a valuable advantage to evade the host immune system. Almost every isolate from unrelated patients appears to have a unique “fingerprint”. Moreover, individuals can be colonized with multiple strains, and strains have been shown to evolve during chronic colonization. Not only does almost every infected person carry his/her own individual *H. pylori* strain, but strains can undergo genetic alteration *in vivo*, driven by an elevated mutation rate and an extensive exchange of genetic material [9,10], leading to free recombination of *H. pylori* genes. These phenomena result in the plasticity of the expression profile of *H. pylori*, also affecting surface proteins such as the outer membrane proteins (OMPs) [11], and suggest that *H. pylori* rapidly adapts to individual hosts; for review see [12] and references cited therein. In fact, it was recently demonstrated that short-term mutation rates in *H. pylori* could be quite fast, partially overlapping with those of viruses [13], with each strain of these hypermutable bacteria acting as a quasispecies [14]. The genetic variability of *H. pylori* affects housekeeping genes, virulence genes, lipopolysaccharide (LPS) and numerous OMP encoding genes, thus contributing to host adaptation, persistence and immune response evasion, giving rise to chronic inflammatory reactions. This review presents the recent progress in characterizing the role of the various Lewis-like antigens and adhesins in the interaction with host cell factors. The role of these interactions in bacterial colonization and pathogenesis is discussed.

## 2. *Helicobacter pylori* Adherence-Associated Molecules

### 2.1. Lipopolysaccharide (LPS)

LPS is the major component of the bacterial cell wall of Gram-negative bacteria. It is an organic compound found in the outer leaflet of outer membranes which contributes to the structural integrity of the bacteria and protects the membrane. Similar to other Gram-negative bacteria, the LPS of *H. pylori* is essential for the bacteria’s survival. The LPS of *H. pylori* consists of an O-specific polysaccharide chain, a core oligosaccharide, and a lipid part called lipid A, embedded in the outer membrane. While LPS is often highly toxic for the host, that of *H. pylori* is low in activation of the host immunological responses [15].

The O-antigen of *H. pylori* LPS contains different human Lewis-like antigens, including Lewis (Le)^x^, Le^y^, Le^a^ and Le^b^, which are also expressed in gastric epithelial cells [16]. It was hypothesized that these *H. pylori* LPS Lewis-like antigens could play a role in adherence to gastric epithelial cells in a in a Lewis-antigen-dependent manner, more specifically via Le^x^. Moreover, since they undergo phase variation and antigenic variation within a single strain, this would provide the bacteria with a dynamic adherent phenotype [17,18,19]. Several studies support the role of LPS as an adherence factor of *H. pylori*. In one study, a monoclonal antibody that inhibited *H. pylori* adherence to gastric epithelial cells by up to 75% was shown to target the LPS possibly through the O-antigen Le^x^ portion [20]. More recently, the receptor recognized by the O-antigen side-chain of *H. pylori* LPS was identified as being a host β-galactoside-binding lectin, galectin-3 [21]. This study also showed that expression of galectin-3 is up regulated by gastric epithelial cells following adherence of *H. pylori*, suggesting that, in addition to colonization, this lectin also plays a role in the host response to infection.

Data from other studies indicate however that Lewis-like antigens seem to only have a limited role in bacterial adherence, which is most likely overcome by the strong adherence phenotype mediated by *H. pylori* adhesins [22,23].

### 2.2. Outer Membrane Proteins

**Figure 1:**
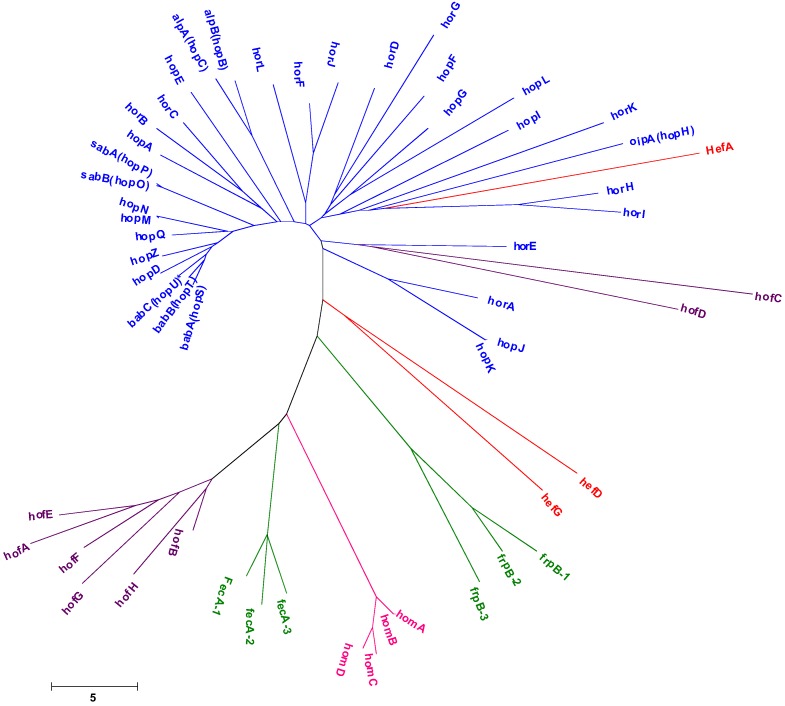
Phylogenetic analysis of the large the set of outer membrane proteins identified in *Helicobacter pylori.*

The OMP profile of *H. pylori* strains differs significantly from that of other Gram-negative species as no major OMPs predominate, rather multiple lower-abundance OMPs are observed [24]. Approximately 4% of the *H. pylori* genome encodes an extraordinary large set of OMPs (~64 OMPs) divided into five paralogous gene families [24] (Figure 1) and this unusual set of OMPs may be a reflection of the adaptation of *H. pylori* to the unique gastric environment where it is found. The largest family is Family 1, comprised of the Hop (for *H. pylori* OMP, 21 members) and Hor (for Hop related, 12 members) proteins. Families 2 and 3 comprise the Hof (for *Helicobacter* OMP, 8 members) and Hom (for *Helicobacter* outer membrane, 4 members) proteins, respectively. Families 4 and 5 are composed of iron-regulated OMPs (6 members) and efflux pump OMPs (3 members), respectively. Other OMPs (~10 members) were not included in these families [24].

Members of the Hop family share highly similar or identical sequences at their amino and carboxyl termini and include porins [25,26] and several known or predicted *H. pylori* adhesins which promote binding to the gastric epithelium [27,28]. In addition, another OMP from the third family, HomB, was shown to be involved in *H. pylori* adherence [29].

The level of the expression of *H. pylori* OMPs can be altered and regulated by several mechanisms, of which the most important are: gene conversion and gene duplication, regulation by phase variation and allelic variation. Several examples of these mechanisms are described in the paragraphs below.

#### 2.2.1. BabA

The major *H. pylori* adhesin is the blood group antigen-binding adhesin A, named BabA or HopS or OMP28 (~80 kDa), which was the first identified adhesin in *H. pylori*; for review see [30,31] and references cited therein. BabA, mediates the binding of the bacterium to the fucosylated Lewis b blood group antigen, Le^b^ [27,32] and related terminal fucose residues found on blood group O (H antigen), A and B antigens [33] which are expressed on the surface of mucins (MUC1 and MUC5B) and gastric epithelial cells, Le^b^ being the dominant antigen in the gastric mucosa [34]. BabA binds to blood group determinants on both type 1 and type 4 core chains [35]. BabA adhesin also binds to the salivary mucin MUC5B, a proline-rich glycoprotein, and to the non-mucin glycoprotein gp-340 [36,37] (Table 1).

The five major families of outer membrane proteins (OMP) previously reported [24] are represented. The OMP gene sequences provided from the *H. pylori* strain J99 [24], except for the *bab*C gene (absent in the J99 strain) provided from the 26695 strain [24]. Family 1 (blue) comprises 21 members named Hop (21 members) and Hor (12 members) proteins. Families 2 (red) and 3 (pink) comprise the Hof (8 members) and Hom (4 members) proteins, respectively. Families 4 (green) and 5 (red) are composed of iron-regulated OMPs (6 members) and efflux pump OMPs (3 members), respectively. The 10 other non-classified OMPs are not included in the phylogenetic tree [24]. The duplicated Hop proteins (HopJ/K and HopM/N) were not differentiated and are each visualized as one line. Neighbour-joining phylogenetic tree topologies of *H. pylori* OMP genes were constructed using the MEGA (Molecular Evolutionary Genetics Analysis) 5.1 software [38], on the basis of distances estimated using the Maximum Composite Likelihood model [39]. Branching significance was estimated using bootstrap confidence levels by randomly resampling the data 1,000 times with the referred evolutionary distance model. The branch length index is represented below the tree. * *bab*C gene sequence provided from the *H. pylori* 26695 strain. Hop, *Helicobacter pylori* OMP; Hor, Hop related; Hof, *Helicobacter* OMP, Hom, *Helicobacter* outer membrane.

**Table 1:** Helicobacter pylori adhesins and their receptors.

Adhesins	Receptors identified	References
BabA	fucosylated Lewis b histo-blood group antigen, Leb and H1	[27,32]
BabA	terminal fucose residues on blood group O (H antigen), A and B antigens	[33]
BabA	salivary nonmucin glycoprotein gp-340	[37]
BabA	salivary mucin MUC5B * and proline-rich glycoprotein	[36,37]
SabA	sialyl-dimeric-Lex	[28]
SabA	salivary mucin MUC7, MUC5B *	[36,37]
SabA	salivary glycoproteins (carbonic anhydrase VI, secretory component, heavy chain of secretory IgA1, parotid secretory protein and zinc-α2-glycoprotein)	[37]
SabA	sialylated moieties on the extracellular matrix protein laminin	[36]
SabA	sialylated structures on the surface of erythrocytes	[40]
SabA	sialylated carbohydrates on neutrophils	[41]
AlpA	laminin	[42,43]
AlpB	laminin	[42,43]

An additional unidentified bacterial surface component (different from BabA and SabA) also mediates the binding to unknown receptor moieties of fibronectin and lactoferrin [36]; * MUC5B carries both fucosylated blood group antigens and α2–3-linked sialic acids.

BabA adhesin has closely related paralogs (Figure 1), BabB (HopT or OMP19) and BabC (HopU or OMP9), whose function have not yet been determined. All of these genes present extensive 5' and 3' homology, in particular *bab*A and *bab*B [44], suggesting that the middle variable region of BabA most likely encodes the specific adhesin function. Five different allele groups of *bab*A (AD1 to AD5) and three different allele groups of *bab*B (BD1 to BD3) were observed within this variable region, none of these alleles being a determining factor in Le^b^ binding of *H. pylori* strains [45]. Phylogenetic analysis revealed that the allelic groupings of *bab*A and *bab*B are independent of one another and that, for both, geographic variation is present [45]. Although *bab*A/*bab*B 5' and middle regions showed strong interstrain similarity, the 3' segments revealed strong intrastrain similarity, indicative of concerted evolution. This segment also demonstrates geographic clustering among *H. pylori* strains [44]. BabA expression is regulated both at transcriptional and translational levels [44,46,47,48,49,50,51]. *bab*A, *bab*B and *bab*C paralogs can be present at multiple loci, with *bab*A and *bab*B usually present at loci A and B, respectively, while the locus C is rarely occupied [46,52,53]. Gene conversion (see Section 3 below) frequently occurs between the loci A and B, most likely by RecA-dependent intragenomic recombination between complementary loci [44] leading to chimeric *bab*B*/*A and *bab*A*/*B genes [46,47,48,49]. For example, non Le^b^ binding strains can regain Le^b^ binding activity by recombination into the *bab*B locus leading to the BabB/A chimera able to bind Le^b^ [47]. *In vivo* loss of BabA expression and Le^b^ binding was also reported in animal models [48,50,51]. The loss of BabA adherence is due to (1) the modification of six amino acid residues critical for the binding to fucosylated blood group antigens [50], (2) nucleotide(s) substitution, deletion (up tp 84 bp) or insertion (1 bp) within various portions of the *bab*A gene resulting in a truncated BabA translation product [51] or (3) phase variation through a slipped strand mechanism (SSM) based on the number of Cysteine-Threonine (CT) dinucleotide repeats in the 5' region of the gene [27,46,48,50]. These CT repeats are probably the result of chromosomal translocation events. Initially, two *bab*A “alleles” were reported; *bab*A2 allele is expressed and involved in the Le^b^ binding activity, while the rarest *babA1* is not expressed due to a 10 bp deletion in its signal peptide sequence [27] that eliminates its translational initiation codon and also due to the presence of four additional nucleotides between the 10- and 35-sites of the *bab*A1 promoter, diminishing the strength of the promoter [47].

Both *in vitro* and *in vivo* experiments revealed that BabA-mediated adherence to Le^b^ on the epithelial surface is a potentiator of the *H. pylori* T4SS secretion system activity, thus triggering the transcription of genes which enhance inflammation, development of intestinal metaplasia, and associated precancerous transformations [54]. BabA binding to Le^b^ is also important for the induction of DNA double-strand breaks and a DNA damage response in host cells in a manner independent of VacA, γ-glutamyl transpeptidase and the *cag* PAI [55].

The presence of *bab*A is associated with *cag*A and *vac*A toxigenic s1 alleles. However, most PCR-based methods currently used to determine the *bab*A status do not evaluate the *bab*A transcriptional regulation mechanisms (see above) that could result in loss of BabA adherence [56]. A few studies evaluated the BabA status by immunoenzymatic-based methods and Le^b^ binding activity. They nevertheless confirmed that the BabA-positive status of infecting strains is associated with CagA and VacAs1 and the presence of intestinal metaplasia and degenerative alterations in human biopsies [57]. BabA-positive status is also closely associated with severe clinical outcomes [58]. Surprisingly, a small group of strains producing low levels of BabA and lacking Le^b^ binding activity were more likely to be associated with increased mucosal inflammation and severe clinical outcomes than BabA-positive strains with Le^b^ binding activity [56]. The underlying mechanism remains unclear and further studies are necessary to investigate the functional coordination of the BabA-receptor network during interaction of *H. pylori* with the gastric epithelium [30].

#### 2.2.2. SabA

The sialic acid-binding adhesin, SabA or HopP or OMP17 (~70 KDa), is the second most well characterized adhesin of *H. pylori*; for review see [59] and references cited therein. Upon *H. pylori* infection and the resulting mucosal inflammation, the sialyl-dimeric-Lewis x (Le^x^) glycosphingolipid is upregulated and SabA mediates the binding of the bacterium to the sialyl-dimeric-Le^x^ [28]. SabA adhesin also participates with BabA in the binding of *H. pylori* to a salivary mucin MUC5B, but to a lesser extent [36,37] (Table 1).

SabA has a closely related paralog, SabB (HopO, OMP16) [24] (Figure 1). The 5' and 3' ends of the *sab*A gene share the highest nucleotide identity with two genes, *sab*B [24] and another OMP, *hopQ* (OMP27) [60], whose function has not yet been determined; both may also be involved in *H. pylori* adherence [61,62]. Variability in copy number and locus of *sab*A and its paralogs, *sab*B and *hop*Q, also occurs among clinical *H. pylori* isolates [60]. Gene conversion is a mechanism for *H. pylori* to regulate the amount of SabA on the bacterial surface and adherence to mouse gastric tissue. Gene conversion mainly results from intragenomic recombination and occurs *in vitro* at a similar rate at both the *sab*A and *sab*B loci but at a lower rate at the *hop*Q locus. The pH appears to be another regulation mechanism of *sab*A expression. Indeed, *sab*A mRNA and SabA expression levels are decreased under acidic conditions [63,64].

The *sab*A gene is one of the most divergent genes in the *H. pylori* genome [65] and is also regulated at transcriptional level by several mechanisms. Indeed, dinucleotide CT repeats present in the 5' coding region of *sab*A regulate its expression by phase variation through SSM [61,64] and the *sab*A promoter region modulates its transcriptional activity through a variable homopolymeric thymidine tract [66]. The frequent “on/off” switch of SabA expression suggests that SabA expression can rapidly respond to changing conditions in the stomach. Molecular-based methods currently used to analyze the CT repeats do not correlate with SabA production defined by immunoblot [59], probably because of the frequent “on/off” switches. SabA positive status was inversely related to the ability of the stomach to secrete acid, suggesting that its expression may be regulated by changes in acid secretion and/or in antigens expressed by the atrophic mucosa [64].

The interaction between SabA and its ligand enhances the colonization density of *H. pylori* in patients lacking gastric Le^b^ [67]. Moreover, *H. pylori* modulates the expression of the SabA ligand, the sialyl-dimeric-Le^x^, in human gastric cell lines via the induction of a specific glycosyltransferase, β3 GlcNAc T5 (β3GnT5), involved in the biosynthesis of Lewis antigens, thereby strengthening the epithelial attachment necessary to achieve successful colonization [68].

SabA has also been identified as a haemagglutinin which binds to sialylated structures found on the surface of erythrocytes, and there is a good correlation among strains between sialic acid dependent haemagglutination and sialyl Le^x^ binding [40]. A high level of polymorphism in sialyl binding properties, similar to that observed for BabA, was reported among clinical *H. pylori* isolates, which suggests that SabA adapts to its host depending on the mucosal sialylation pattern of the infected individual [55].

Arrays and proteomic studies revealed that SabA also mediates the binding of *H. pylori* to sialylated moieties on the extracellular matrix protein laminin [36] and to salivary glycoproteins, including MUC7, carbonic anhydrase VI, secretory component, heavy chain of secretory IgA1, parotid secretory protein and zinc-α2-glycoprotein [37]. SabA was also found to interact with BabA in complexes that could be potentially implicated in the development of malignant diseases [69].

SabA-positive status was associated with gastric cancer, intestinal metaplasia, and corpus atrophy in Western strains [61] but not in Asian strains [67], suggesting differences between SabA and clinical outcome. Upon *H. pylori* induced gastritis, neutrophils and monocytes infiltrate into the gastric mucosa. SabA of nonopsonized *H. pylori* strains specifically binds to neutrophils through sialylated carbohydrates. As a consequence, the stimulated neutrophils produce reactive oxygen species causing oxidative damage of the gastric epithelium, demonstrating that SabA is nevertheless a true virulence factor [41].

#### 2.2.3. AlpA, AlpB

The adherence associated lipoproteins AlpA (HopC or OMP20, ~56 kDa) and AlpB (HopB or OMP21, ~57 kDa) are encoded by highly homologous adjacent genes, *alp*A and *alp*B [24,70]. Both lipoproteins were found interacting in a *H. pylori* membrane complex [69,71] and are involved in gastric colonization [72]. Indeed, the corresponding mutants did not infect the stomachs of guinea pigs and Mongolian gerbils [73,74] and poorly colonized the stomachs of C57BL/6 mice [75]. AlpA and AlpB are essential in the adherence of the bacterium to human gastric tissue in a different pattern than that observed for the BabA-mediated adherence, suggesting that a different receptor may be involved [42]. Both lipoproteins contribute to host laminin binding and influence gastric inflammation in gerbils; the expression of plasmid-borne *alp*A or *alp*B confers laminin-binding ability to *Escherichia coli* [43]. No other host binding partner or receptor has yet been identified. In contrast to many other OMPs that are produced at extremely variable rates, AlpA and AlpB proteins are strongly coproduced and seem to be expressed by all clinical isolates, supporting their essential function [11]. The protein expression level of AlpA is nevertheless upregulated in response to oxidative stress [76]. AlpA and AlpB induce gastric injury by modulating proinflammatory intracellular signalling cascades upon infection [43]. However, discrepant results regarding the inflammatory effect of the *alp*AB locus were obtained after infection of mice and gerbils [43,75]; the AlpA-AlpB induced interleukin-8 (IL-8) secretion seems to be related to the isolate’s geographic origin [75]. The fact that both these proteins seem to be constitutively expressed by all *H. pylori* isolates and are recognized specifically by sera from *H. pylori*-infected patients [71,77] makes these proteins attractive targets for the vaccine design [78,79].

#### 2.2.4. OipA

The outer inflammatory protein A (OipA, HopH or OMP13 ~34 kDa) was initially identified as a surface protein which promotes *in vitro* IL-8 secretion in a T4SS-independent manner and heightens gastric inflammation *in vivo* [80,81]. OipA regulates IL-8 secretion through PI3K/Akt and this regulation is dependent on FoxO1/3a inactivation [82]. However, the role of OipA in inflammation is controversial. Indeed, *hop*H mutagenesis did not alter epithelial IL-8 secretion *in vitro* [83] or inflammation in Mongolian gerbils [84]. While the OipA host receptor remains unknown, predicted exposed amino acid sequences were determined *in silico* [85] and will be useful in finding its receptor(s). Indeed, there is no doubt that OipA is involved in bacterial adherence to gastric cells *in vitro* [83]. The *hop*H gene does not have highly similar paralogs and is quite distant from other *hop* genes (Figure 1). OipA expression is regulated by phase variation within a CT dinucleotide repeat motif located in the 5' region of the *hop*H gene [80,86]. OipA “on”-status is significantly associated with more severe gastric diseases (duodenal ulcer and gastric cancer), high *H. pylori* density, and severe neutrophil infiltration [53,64,84,87], probably because of its association with other virulence factors or increased bacterial adherence and colonization [83]. The strong link between the OipA “on”-status and *cag*A suggests that OipA may contribute to the fitness of *cag*A-positive strains *in vivo* [53,83]. OipA is involved in *H. pylori*-induced focal adhesion kinase activation and cytoskeletal reorganization of gastric epithelial cells in the early stages of *H. pylori* infection [88]. Isogenic *oip*A mutants significantly reduce the phosphorylation of paxillin, a focal adhesion associated adaptor protein involved in the regulation of *H. pylori*-mediated gastric epithelial cell motility, and also actin stress fiber formation [89]. Inactivation of *oip*A also results in a decreased level of nuclear β-catenin *in vitro* and a reduced incidence of cancer in gerbils [84], indicative of the importance of this OMP in the virulence of *H. pylori*. Vaccination trials based on oral therapeutic immunization using OipA-DNA-based vaccines conferred effective protection against *H. pylori* challenge in mice with a strong Th1/Th2 immune response, eliminating *H. pylori* colonization in the stomach [90,91].

#### 2.2.5. HopZ

The *hop*Z (*omp1*) gene encodes a 74 kDa-protein (681 residues) located on the surface of the bacteria [92]. Immunofluorescence revealed that the HopZ variable region (amino-acids 126 to 281) is exposed. HopZ involvement in adherence was demonstrated *in vitro* as a *hop*Z-knockout mutant strain showed significantly reduced binding capacity to the gastric epithelial AGS cell line, compared to the corresponding wild-type strain [61]. No receptor could be identified for this putative adhesin. *In vivo* the role of the HopZ protein in adherence and colonization is not clear. Indeed, lack of production of HopZ did not affect the ability of the bacteria to colonize the stomachs of guinea pigs [73], while genetic inactivation of *hop*Z reduced the ability of *H. pylori* to survive in the stomachs of gnotobiotic transgenic mice [93]. Two allelic variants (I and II) were reported for the *hop*Z gene [91] and recombination events occurred and led to the exchange of allele fragments [94]. Although *hop*Q (OMP27) is the closest to *hop*Z gene (Figure 1), no recombination event between these loci has been reported to date.

The expression of the *hop*Z gene is regulated by SSM within CT dinucleotide repeats of different length localized at the 5' end of the coding region [92]. The HopZ switch status influenced both *H. pylori* density and its colonization ability in mice [61]. The *hop*Z “off” status frequently switches to “on” during human infection [95] and this status selected during early infection remains subsequently stable, suggesting a strong *in vivo* selection for this putative adhesin during early colonization [94]. The expression status of *hop*Z does not seem to correlate with the disease outcome, except for MALT lymphoma associated strains that appear to express a significantly smaller amount of the protein [95,96,97]. The *hop*Z “off” status was indeed associated with the development of MALT lymphoma [98].

#### 2.2.6. HomB

The smallest family of OMP is the Hom family constituted of four members. The most studied gene is *hom*B encoding the HomB protein (~75 KDa) expressed in the *H. pylori* outer membrane [29]. *hom*B has a closely related paralog, *hom*A (Figure 1), sharing 90% sequence identity, especially at the 5' and 3' ends with the differences between the two confined to the middle region of the respective open reading frames [24]. Allelic diversity was observed in the middle region of the genes, with six different alleles currently identified and expressed *in vivo* [99]. HomA/B variants likely occur via the accumulation of single nucleotide polymorphisms. No correlation between any allele and disease outcome was observed. For each gene, a dominant worldwide allele was detected (allele *AII* for *hom*A and allele *AI* for *hom*B), suggesting that *hom*A/*hom*B allelic variants are independent of the geographical origin of the strain. It was shown by an *in silico* approach that the most prevalent *AI* and *AII* alleles result from a homologous recombination between the rarest allelic variants of each gene, with a crossover point localized in the middle of the genes, containing the allelic region [100]. As for *bab*A/*bab*B, segment 3 (3' end) of *hom*A/*hom*B is under concerted evolution, in contrast to segment 1 (5' end) which displays a divergent evolution [36,96]. *hom*A/*hom*B can also be present at multiple well conserved loci [24,99,101] and, although diversity regarding the number of copy does exist, there is a clear geographical specificity, suggesting an involvement of these genes in host adaptation. Furthermore, *hom*A and *hom*B sequence analysis also suggests regulation by phase variation [99].

**Figure 2:**
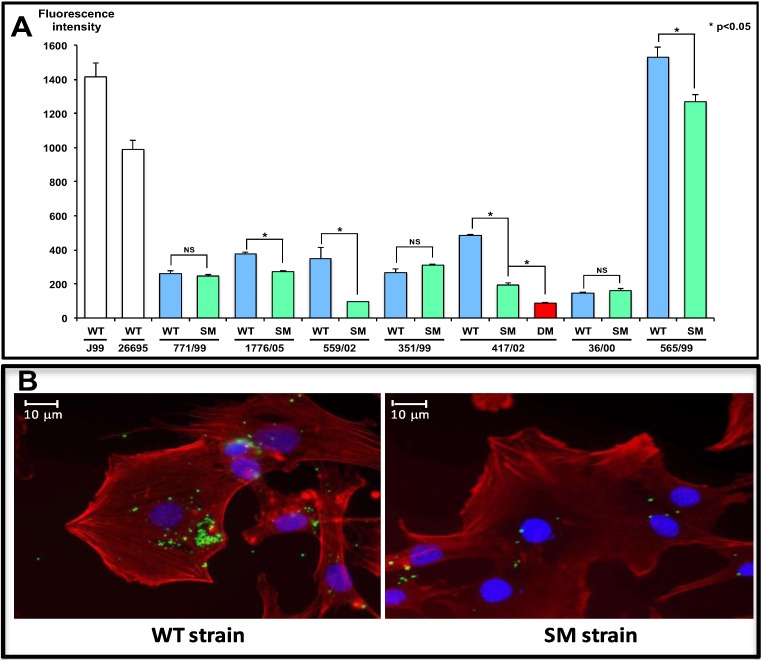
Involvement of the outer membrane protein HomB of *Helicobacter pylori* in interleukin 8 secretion and adherence to gastric epithelial AGS cells.

HomB is present in the membrane of *H. pylori* and is antigenic in humans [29]. Moreover, the protein is associated with IL-8 secretion *in vitro* and contributes to bacterial adherence, both of these functions being correlated with the number of *hom*B copies present in a strain [29] (Figure 2). In contrast to *hom*A, *hom*B is associated with the presence of *cag*A [29,102,103,104], as well as inflammation and atrophy in the corpus [103]. It clearly appears that *hom*B is a marker of more virulent *H. pylori* strains, which influences the severity of disease and contributes to more severe clinical outcomes. However, the exact involvement of HomB in the duodenal ulcer or gastric cancer outcome is not completely elucidated and is likely to be influenced by the geographical origin of the isolate [105,106]. Despite HomB’s role in adherence, a host receptor remains to be determined and will be a major challenge for future research.

Cells were infected for 18 h with *H. pylori* clinical strains, wild-type (WT) and *hom*B knockout mutant strains, single mutant (SM) and double mutant (DM) strains, at a multiplicity of infection of 100. (**A**) Adherence of *H. pylori* was determined by flow cytometry after bacteria labelling using the PKH2 green fluorescent linker kit (Sigma). Values denote the means of the triplicate (±SD). (Adapted from [29]). Two sequenced *H. pylori* strains 26695 (ATCC 700392) which were positive for *hom*A and negative for *hom*B, and J99 (ATCC 700824) which was positive for both *hom*A and *hom*B were used as reference strains [70,107]. Seven *H. pylori* clinical strains were used. The latter strains were all *hom*A negative. The strain 417/02 contained 2 copies of *hom*B at the *hom*A and *hom*B locus (GenBank accession nos. EF648377 and EF648378, respectively). All of the other 6 strains carried a single copy of *hom*B at the *homB* locus (strains 771/99, 1776/05, 559/02, 351/99, 36/00 and 565/99 (GenBank accession nos. EF648357, EF656577 to EF656579, EF648369 and EF648370). DM, double mutant; NS, not significant; SM, single mutant; WT, wild-type. (**B**) Adherence of *H. pylori* wild-type (WT) and single *hom*B knockout mutant (SM) strains to AGS cells. AGS cells were processed for fluorescent staining with fluorescent labelled-phalloidin to detect F-actin (red) and Hoechst 33342 compound to detect the nucleus (blue). Primary antibody followed by fluorescent labelled-secondary antibodies (green) was used to detect *H. pylori*.

## 3. Major Gastric Epithelial Receptors of *H. pylori*

*H. pylori* exhibits both host tropism, exclusively colonizing humans and some primates, and tissue tropism, adhering only to the gastric epithelial lining of the antrum or staying in the gastric mucus layer. In the duodenum, gastric metaplasia is a pre-requisite for the presence of the bacterium, and reports exist of *H. pylori* colonization at distant sites of gastric metaplasia, such as in Meckel’s diverticula containing gastric mucosa and in the rectum [108,109]. In addition, *H. pylori* is not able to colonize areas of complete intestinal metaplasia in the gastric mucosa [110]. The majority of the bacteria remain in the mucus layer, with a smaller proportion colonizing the gastric surface. 

### 3.1. Lewis Blood Group Antigens

*H. pylori* adheres to the mucus-secreting gastric epithelial cells of the lumen in the upper half of the gastric pit, but not to closely related mucus-secreting neck cells in the lower half of the gastric pit. *H. pylori* also does not adhere to chief cells, parietal cells, or endocrine cells in the gastric pit.

The major component of the gastric mucus layer are the highly glycosylated mucin proteins. In a healthy gastric mucosa, the human mucins (MUC) produced include MUC1, MUC5AC and MUC6. The membrane associated MUC1 is expressed in foveolar cells; the secreted MUC5AC mucin, a major constituent of the surface mucus gel layer, is restricted to the foveolar epithelium; and finally the expression of the secreted MUC6 is limited to the neck cells of the glands. This mucin distribution establishes the gastric glycosylation pattern as the expression of MUC5AC is accompanied by a similar distribution of fucosyltransferases, leading to co-expression of type 1 antigens, Le^a^ and Le^b^ and H-type 1 blood group antigens. Blood group antigens are a group of carbohydrates typically found on erythrocytes, and also highly expressed in epithelial tissues, including the gastric mucosa.

The fucosylated H-type 1 and Le^b^ antigens are naturally expressed on the gastric mucosa of secretor or Lewis-positive individuals. The secretor status depends on an active α(1,2)-fucosyltransferase that catalyses the addition of terminal α(1,2)-fucose residues. On the other hand, MUC6 expression is associated with the type 2 antigens, Le^x^ and Le^y^, which are consequently located deeper in the glands, e.g., in the mucus, chief and parietal acid-producing cells [111,112].

Studies show that *H. pylori* is very closely associated with extracellular MUC5AC and epithelial cells that produce MUC5AC, indicating that MUC5AC, but not MUC6, plays a role in the adherence of *H. pylori* to the gastric mucosa [113]. In addition MUC6 exhibits an antimicrobial activity [114]. The major receptor for *H. pylori* is the Le^b^ carbohydrate structure present in the normal gastric tissue and MUC5AC is the most important carrier of Le^b^, with the attachment being made through BabA [115]. 

Several studies demonstrated that the glycosylation pattern of gastric mucins differs between individuals and changes during gastric disease evolution within an individual, implying that *H. pylori* has to constantly adapt to these environmental modifications. A recent study evaluated the effect of glycan expression modifications in *H. pylori* adherence, by using the animal model of non-secretors, Fut2-null mice, which exhibit a reduced expression of α(1,2) fucosylated structures on their gastric mucosa. It was shown that strains expressing a functional BabA adhesin showed decreased adherence to the gastric mucosa of the Fut2-null mice, while the binding pattern of strains expressing only the sialic acid-binding adhesin SabA was not altered, demonstrating that the impaired adherence could be attributed to reduced expression of BabA ligands in the gastric mucosa of Fut2-null mice [116].

### 3.2. Sialylated Glycans

All *H. pylori*-infected individuals develop a chronic gastritis, with extensive inflammation of the mucosa, with a small fraction developing pre-neoplastic alterations such as atrophic gastritis and in a later stage intestinal metaplasia [117]. Under these clinical conditions, Le^b^ is weakly expressed while there is an upward migration of Le^x^ associated with atrophy of the epithelium. Moreover, inflammation of the gastric mucosa leads to the expression of sialylated glycans, such as sialyl-Le^a^ and sialyl-Le^x^. In these cases, *H. pylori* will bind to sialyl-Le^x^ via SabA adhesins [28]. Accordingly, it was demonstrated that SabA interaction with the host gastric sialyl-Le^x^ antigen enhanced *H. pylori* colonization in patients with weak or no Le^b^ expression [67].

## 4. The Importance of Adherence for Bacterial Colonization and Virulence

*H. pylori* is very well adapted to its host and persists for decades in the human stomach. During an established infection, some of the *H. pylori* cells attach themselves directly to epithelial cells, while the vast majority of the cells (~70%) are found in the mucus layer of the superficial gastric mucosa, either motile or adhering to the heavily glycosylated secreted mucins. This location favours the acquisition of nutrients released from the damaged host cells and delivery of effector molecules into the gastric cell, such as the CagA oncoprotein and the VacA cytotoxin, while at the same time bacteria are prevented from being eliminated from the stomach by mucus turnover and gastric peristalsis. However, the tight adherence of *H. pylori* to the mucosa may be deleterious when host inflammatory and immune responses are robust. Thus, the bacteria must be able to manage, in a fast and effective way, the delicate balance between the adherent and the motile population.

The importance of adherence to the colonization of the gastric niche has been highlighted by several studies showing a positive correlation between BabA-mediated adherence to Le^b^ antigens and an increased bacterial colonization. Indeed, patients with gastric Le^b^ expression had a higher bacterial density than those without Le^b^ expression [118,119].

Even so, as explained previously, *H. pylori* adherence is not solely mediated by BabA-Le^b^ interaction, as it can also be mediated by Lewis antigens other than Le^b^, such as Le^a^, Le^x^ and its sialylated form, most likely via other adhesins [119]. Added proof is the fact that *H. pylori* can establish a chronic infection in both weak-secretor and non-secretor individuals as well, with these phenotypes estimated to affect nearly 20% of the human population [57,120,121]. In line with this, a study showed that BabA and SabA-negative strains could bind to gastric tissue in an AlpAB-dependent manner [22].

**Figure 3:**
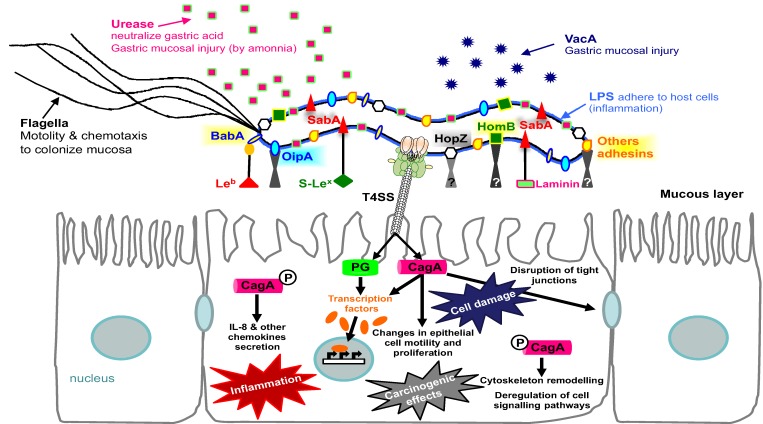
The role of bacterial adherence in *Helicobacter pylori* virulence.

Several studies showed that adherence of *H. pylori* to the gastric mucosa plays a pivotal role in the outcome of infection. Indeed, the attachment of the bacterium to the epithelial cells induces signal transduction pathways in gastric cells, with activation of the transcription factor nuclear factor-κB. Subsequently, the cytokine IL-8 (an important T cell and neutrophil chemoattractant, and critical mediator in the *H. pylori* induced inflammatory process) is produced. One mode is through BabA-mediated binding to the host cells, which enables translocation of CagA, triggering a strong cytokine response. Moreover, once translocated, CagA activates numerous host signaling pathways, inducing epithelial responses with carcinogenic potential, for a review see [122,123] and references cited therein (Figure 3).

In the mucous layer, *H. pylori* binds to the apical membrane of gastric epithelial cells via BabA, SabA and other adhesins. The role *H. pylori* LPS in adherence to gastric epithelial cells is still unclear [19,20,21,22]. *H. pylori* strains expressing a type IV secretion system (T4SS) encoded by the *cag* pathogenicity island are known to upregulate transcription factors such as the nuclear factor (NF)-κB, leading to production of pro-inflammatory cytokines and chemokines. Once adherent, *H. pylori* assembles T4SS pili (a needle-like pilus device) at their surface enabling the translocation of bacterial effector proteins into the cytoplasm of host gastric epithelial cells, such as the CagA oncoprotein and peptidoglycan. The intracellular delivery of CagA activates numerous host signaling pathways, inducing epithelial responses with carcinogenic potential. Le^b^, fucosylated Lewis b blood group antigen; S-Le^x^, sialyl-dimeric-Lewis x glycosphingolipid; LPS, lipopolysaccharide; P, phosphorylated; PG, peptidoglycan.

The biological importance of the adhesin BabA-Le^b^ receptor interaction on the pathogenic features of *H. pylori* has been highlighted in several studies [56,124]. Recently, it was shown both *in vitro* and *in vivo* that BabA-mediated binding of *H. pylori* to Le^b^ on the epithelial surface increases T4SS-dependent *H. pylori* pathogenicity by triggering the production of proinflammatory cytokines CCL5 and IL-8 and the precancer-related factors, CDX2 and MUC2 [54].

The role of *H. pylori* attachment in the determination of clinical outcome has also been demonstrated by the epidemiological link between adherence-encoding genes and virulence determinants. In Western countries, clinical strains harbouring the triple genotype *bab*A, *cag*A and the most virulent allele of *vac*A, the s1 allele, have been associated with duodenal ulcer and gastric adenocarcinoma [58,124,125]. The correlation of *H. pylori* adherence with inflammation and more severe disease outcome was also demonstrated for another less well studied OMP, HomB [102,103,104]. *In vitro* co-culture assays on gastric epithelial AGS cells revealed that HomB contributes to bacterial adherence and induces activation of IL-8 secretion, depending on the number of *hom*B copies present in a strain (Figure 2) [29].

A recent study explored how *H. pylori* is affected by the host, in particular by the host mucins, and what impact this regulation may have on the clinical outcome. The authors showed that mucin binding ability via BabA was an important factor for enhancing the *H. pylori* proliferation, with higher proliferation rates being observed with tumor-derived mucins and mucins from the surface mucosa, consisting mostly of MUC5AC, than with gland-derived mucins consisting mainly of MUC6 [126]. In addition, they showed that, in an adherence dependent manner, *H. pylori* responded to the presence of mucins with an upregulation of genes involved in colonization (*bab*A, *sab*A, *fla*A and *ure*A) and virulence processes (*cag*A), suggesting once again that these genes are co-regulated in response to mucins. Therefore, the authors hypothesized that regulation of *H. pylori* by mucins may be an important explanation for individual variations in host response and symptoms following infection [126].

Besides the interaction of BabA adhesin and structural T4SS proteins in causing gastric lesions through CagA injection, it was shown, using the mouse model of infection, that the attachment of *H. pylori* to Le^b^ led to an increase in the production of auto-antibodies to the host Le^x^ antigens, produced either in bacterial LPS and in the acid-secreting parietal cells [127]. These data also suggests a significant impact of *H. pylori* adherence on the development of gastritis and gastric atrophy.

## 5. Conclusions

The gastric pathogen *H. pylori* has colonized the human stomach for more than two hundred thousand years and, despite a currently declining infection in developed western countries, it continues to spread among humans, with high incidence in developing countries in Asia and Africa. This unequivocal success as a pathogen/colonizer is due to complex strategies to maintain an inflammation of the gastric epithelium while limiting the extent of the immune response in order to prevent its elimination. One of the mechanisms contributing to an active host adaptation is without a doubt its adherence to the mucus layer and to the gastric epithelial cells, a complex phenotype involving multiple adhesins and different receptors whose availability changes according to inflammation and glycan expression at the epithelial surface. These molecules are all part of the vast repertoire of OMPs displayed by *H. pylori* strains, which are subjected to different mechanisms that alter and regulate their expression, therefore playing a role in the colonization of the bacteria, persistence of infection and ultimately in the severity of the associated disease. *H. pylori* adherence is far from being clear and future work should be carried out in search of additional adhesins as well as in clarifying the complex adherence-receptor network. Ultimately, these data should provide novel bacterial therapeutic or vaccine targets for the eradication of this persistent bacterium. 
